# NGS analysis in Marfan syndrome spectrum: Combination of rare and common genetic variants to improve genotype-phenotype correlation analysis

**DOI:** 10.1371/journal.pone.0222506

**Published:** 2019-09-19

**Authors:** Davide Gentilini, Antonino Oliveri, Teresa Fazia, Alessandro Pini, Susan Marelli, Luisa Bernardinelli, Anna Maria Di Blasio

**Affiliations:** 1 Department of Brain and Behavioral Sciences, University of Pavia, Pavia, Italy; 2 Istituto Auxologico Italiano IRCCS, Bioinformatics and Statistical Genomics Unit, Cusano Milanino, Milano, Italy; 3 Istituto Auxologico Italiano IRCCS, Molecular Biology Laboratory, Cusano Milanino, Milano, Italy; 4 Rare Disease Center, Marfan Clinic, Cardiology department, ASST-FBF-Sacco, Milano, Italy; 5 Centro di Cardiogenetica Vascolare IRCCS Policlinico San Donato, San Donato Milanese, Milano, Italy; Heart and Diabetes Center NRW, University Hospital of the Ruhr-University Bochum, GERMANY

## Abstract

The diagnosis of Marfan spectrum includes a large number of clinical criteria. Although the identification of pathogenic variants contributes to the diagnostic process, its value to the prediction of clinical outcomes is still limited. An important novelty of the present study is represented by the statistical approach adopted to investigate genotype-phenotype correlation. The analysis has been improved considering the extended genetic information obtained by Next Generation Sequencing (NGS) and combining the effects of both rare and common genetic variants in an inclusive model. To this aim a cohort of 181 patients were analyzed with a NGS panel including 11 genes associated with Marfan spectrum. The genotype-phenotype correlation was also investigated considering the possibility to predict presence of a pathological mutation in Marfan syndrome (MFS) main genes based only on the analysis of phenotypic traits. Results obtained indicate that information about clinical traits can be summarized in a new variable that resulted significantly associated with the probability to find a pathological mutation in MFS main genes. This is important since the choice of the genetic test is often influenced by the phenotypic characterization of patients. Moreover, both rare and common variants were found to significantly contribute to clinical spectrum and their combination allowed to increase the percentage of phenotype variability that could be explained based on genetic factors. Results highlight the opportunity to take advantage of the overall genetic information obtained by NGS data to have a better clinical classification of patients.

## Introduction

Marfan syndrome (MFS) is a genetic condition that affects connective tissues and has wide range of clinical severity, ranging from isolated features [1] to neonatal presentation of severe and rapidly progressive disease involving multiple organ systems [2,3]. Although many clinicians view the disorder in terms of classic ocular, cardiovascular, and musculoskeletal abnormalities [4], manifestations also include involvement of the lung, skin, and central nervous system [5]. A wide variety of skeletal abnormalities occurs with MFS, including arachnodactyly, scoliosis, chest wall deformity and ligamentous laxity [6,7].

There are disorders having a great overlap of clinical signs with MFS and collected under the definition of Marfan-like phenotype [8]: e.g. Loeys-Dietz Syndrome (LDS), Ehlers-Danlos Syndrome [9] (EDS), and Familial Thoracic Aortic Aneurysm and Dissection [10] (TAAD), Ectopia Lentis Syndrome [11] (ELS), Beals Syndrome [12] (BS), Sticler Syndrome [13] (SS), Bicuspid Aortic Valve [14] (BAV) and MASS phenotype [15] (Mitral valve, myopia, Aorta, Skin and Skeletal features of the disorder). The complexity of MFS clinical picture and its unpredictable course were extensively studied in the last decades, especially trying to elucidate the genotype-phenotype correlations of MFS and Marfan-like *habitus*. Mutations of *FBN1* gene, encoding fibrillin-1, represent the most common genetic event associated with Marfan-like spectrum since a *FBN1* mutations can be identified in 91–95% of subjects with classic MFS [16,17]. However, *FBN1* cannot be considered the unique genetic cause of marfanoid *habitus* since there are additional genes known to be associated with other Marfan-like disorders. Among these genes *ACTA2*, enconding actin, *MYH11*, encoding myosin-11, *MYLK*, encoding myosin light chain kinase, *NOTCH1*, encoding neurogenic locus notch homolog protein 1, *SMAD4*, encoding mothers against decapentaplegic homolog 4, are associated with TAAD, while *COL3A1*, encoding collagen alpha-1(III) chain, *COL5A1*, encoding collagen alpha-1(V) chain, *COL5A2*, encoding collagen alpha-2(V) chain, *FLNA*, encoding filamin-A, are known to be associated with EDS, and *TGFBR1*, encoding TGF-beta receptor type-1, *TGFBR2*, encoding TGF-beta receptor type-2, *SMAD3*, encoding mothers against decapentaplegic homolog 3, are reported to be associated with LDS/TAAD. Previous studies that aimed to investigate genotype-phenotype correlation in Marfan-like spectrum focused their attention mainly on *FBN1* pathogenic variants [18,19]. In these studies, mutations were often classified according to their domain localization or to protein truncating or in-frame variants types without considering the effect on the protein product [20,21]. Studies based on these classifications of *FBN1* mutations observed limited genotype-phenotype correlations, with the exception of the association of early onset, severe (previously named “neonatal”) MFS and mutations in exons 24–32 [22], and notably the observation of a higher probability of ascending aortic dilatation, aortic event, and mitral valve prolapse in patients with variants altering a cysteine residue [23]. Moreover, a recent study showed that MFS patients with an haploinsufficient variants have an increased risk of cardiovascular death and aortic dissection compared with patients with a dominant negative variant [24]. Notwithstanding these interesting results, the genotype-phenotype correlation in MFS and Marfan-like disorders still remain largely unexplored. Results from literature suggest that studying only pathogenic variants of *FBN1*, only a small percentage of variability of phenotypic traits and clinical signs can be explained by genetics. The main aim of the present study was to improve the genotype-phenotype correlation by widening the amount of genetic information considered. To this aim we extended genetic analysis to several genes already known to be associated with other Marfan-like disorders. The association analysis between genotypes and phenotypes was performed considering the extended genetic information that can be obtained with Next Generation Sequencing (NGS) technology and combining the effects of both rare and common genetic variants. The possibility to predict the presence of a pathogenic variant in MFS associated genes such as *FBN1* or *TGFBRs* based on the analysis of phenotypic manifestations was also investigated. This issue is extremely important in the contest of Marfan-like syndromes since the genetic test and the gene panel usually selected for the analysis might be influenced by the clinical classification of the patients.

## Materials and methods

### Patients and phenotypic data

A sample of 181 unrelated patients with a Marfan-like phenotypic picture were from “Centro Marfan” of the Luigi Sacco’s Hospital in Milan (Italy). Molecular analysis was performed at the Istituto Auxologico Italiano in Milan (Italy). Inclusion criteria were: patients with family history of aortic disease, early onset of aortic disease, suspected connective tissue disorders, and patients suspected of having MFS, according to 2010 Ghent criteria [25]. Medical and personal history of the patients were recorded. Clinical information included: presence of skeletal signs (arachnodactyly, scoliosis, pectus carinatum, flatfoot, thumb and wrist sign, reduced elbow extension, hyperlaxity), presence of cardiovascular signs (aortic dissection, aortic ectasia, mitral valve prolapse), and oftalmologic signs (ectopia lentis, myopia), presence of cutaneous signs (stretch marks). Systemic score for each patient was calculated with web calculator (http://www.marfan.org/resources/professionals/marfandx). All patients had echocardiographic study and computerized tomography (CT) scan of the entire aorta. Diagnosis for mitral valve prolapse was based upon published criteria [25].

Approval for this study was granted by the local Istituto Auxologico Italiano Ethics Committee, approval number: 2013_06_04. All study participants gave an informed written consent including specific consent to genetic testing and permission to publish the results.

### Gene panel and sequencing experiments

Illumina TruSeq Custom Amplicon Kit was used to capture all exons, intron–exon boundaries and at least 50-bp flanking sequences of target genes (RefSeq database, hg19 assembly). Marfan-like disease panel was composed by 11 genes (i.e, *FBN1*, *TGFBR1*, *TGFBR2*, *COL1A1*, *COL1A2*, *COL3A1*, *COL5A1*, *COL5A2*, *MYH11*, *NOTCH1* and *ACTA2)* known to be associated with MFS, EDS, TAAD, ABV, and marfanoid *habitus*. A description of the examined panel of 11 genes and their exons including chromosomal locus references sequence, protein name and associated disease was summarized in Table 1. The TruSeq Custom Amplicon assay (Illumina, San Diego, CA) was designed by the DesignStudio software, having 545 amplicons covering exons with at least 50 bases of the flanking introns; the total coverage of the target genes by the designed amplicons was > 95%. Genomic DNA of each patient was extracted from peripheral blood lymphocytes using Gene Catcher gDNA 96x10 ml Automated Blood kit (Invitrogen, Life TechnologiesTM). The TruSeq NGS libraries were prepared according to the manufacturer’s instructions (Truseq^®^ Custom Amplicon Library Preparation, Illumina, San Diego, CA). Pooled libraries were sequenced on MiSeq Reagent kit v.3 on an Illumina MiSeq sequencer (Illumina, San Diego, CA).

**Table 1:** Description of the examined panel of 11 genes and their exons including chromosomal locus references sequence, protein name and associated disease.

Gene Name	Cytogenetic location	Genomic coordinates (GRCh37/hg19)	Exon count	Protein	Associated diseases
FBN1	15q21.1	chr15:48,700,503–48,938,046	66	fibrillin-1	Acromicric dysplasia, Ectopia lentis, familial, Geleophysic dysplasia 2, Marfan lipodystrophy syndrome, Marfan syndrome, MASS syndrome, Stiff skin syndrome, Weill-Marchesani syndrome 2, dominant
ACTA2	10q23.31	chr10:88,935,074–88,991,390	10	Actin	Aortic aneurysm, familial thoracic 6, Moyamoya disease 5, Multisystemic smooth muscle dysfunction syndrome
MYH11	16p13.11	chr16:15,703,135–15,857,033	43	myosin-11	Aortic aneurysm, familial thoracic 4
NOTCH1	9q34.3	chr9:136,494,433–136,546,048	34	neurogenic locus notch homolog protein 1	Adams-Oliver syndrome 5, Aortic valve disease 1
COL1A1	17q21.33	chr17:50,183,289–50,201,648	51	Collagen alpha-1(I) chain	Caffey disease, Ehlers-Danlos syndrome, arthrochalasia type, 1, Osteogenesis imperfecta, type I, Osteogenesis imperfecta, type II, Osteogenesis imperfecta, type III, Osteogenesis imperfecta, type IV, Bone mineral density variation QTL, osteoporosis
COL1A2	7q21.3	chr7:94,394,561–94,431,232	52	Collagen alpha-2(I) chain	Ehlers-Danlos syndrome, arthrochalasia type, 2, Ehlers-Danlos syndrome, cardiac valvular type, Osteogenesis imperfecta, type II, Osteogenesis imperfecta, type III, Osteogenesis imperfecta, type IV, Osteoporosis, postmenopausal
COL3A1	2q32.2	chr2:188,974,320–189,012,746	51	collagen alpha-1(III) chain	Ehlers-Danlos syndrome, vascular type, Polymicrogyria with or without vascular-type EDS
COL5A1	9q34.3	chr9:134,641,774–134,844,843	67	collagen alpha-1(V) chain	Ehlers-Danlos syndrome, classic type, 1
COL5A2	2q32.2	chr2:189,031,898–189,225,314	55	collagen alpha-2(V) chain	Ehlers-Danlos syndrome, classic type, 2
TGFBR1	9q22.33	chr9:99,104,038–99,154,192	11	TGF-beta receptor type-1	Loeys-Dietz syndrome 1, Multiple self-healing squamous epithelioma, susceptibility to
TGFBR2	3p24.1	chr3:30,606,493–30,694,142	11	TGF-beta receptor type-2	Colorectal cancer, hereditary nonpolyposis, type 6, Esophageal cancer, somatic, Loeys-Dietz syndrome 2

Data collected from NGS experiments were analyzed in order to identify single nucleotide variants and small insertions/deletions and to obtain a matrix of genotypes (*Plink*-format pedigree file). A collective of genetic data was also obtained from the genome Aggregation Database (gnomAD) [26].

### Bioinformatic data analysis

#### Primary analysis

Data collected from NGS experiments were analyzed in order to identify single nucleotide variants and small insertions/deletions. The first steps (including base calling and demultiplexing) was performed using *MiSeq* provided software (Real Time Analysis RTA v.1.18.54 and Casava v.1.8.2, Illumina, Inc., San Diego, CA). FastQ files for each sample, containing mate paired-end reads after demultiplexing and adapter removal, were used as input for MiSeq pipeline. Brefly, FastQ files were processed with *MiSeq Reporter* v2.0.26 using the Custom Amplicon workflow. This analytical method required FastQ files, a “Manifest file” containing information about the sequences of primer pairs, the expected sequence of the amplicons and the coordinates of the reference genome (Homo sapiens, hg19, build 37.2) as input. Each read pair was aligned using the MEM algorithm of the *BWA* software [27]. The aligned BAM file were used as input to GATK variant caller (Genome Analysis ToolKit, v1.6) [28], thus generating a VCFv4.3 file for each sample. NGS data have been uploaded and are available at the public repository for research data Harvard Dataverse https://doi.org/10.7910/DVN/DEAEVL. All other data are within the paper and its Supporting Information files.

#### Secondary analysis

The second step of the bioinformatic analysis was aimed at obtaining a matrix of genotypes (*Plink*-format pedigree file) starting from classical VCFv4.3 files. A bioinformatic framework able to start from classical VCF files without a genomeVCF files intermediate step was developed to this aim. Brefly, starting from classical VCFv4.3 files the following steps were done: i) a list of all genetic variants identified in the sample population was produced, ii) all the genetic variants in the list were used to create a pedigree file reporting for each subject the zigosity status indicated in the respective vcf file (0/1 or 1/1 for heterozygotes and homozygotes respectively), iii) for each subject all genetic variants in the list but not present in the vcf file were inferred to be wild type (0/0), iv) using the function depth of samtools [29] and bedtools [30] programs a BED formatted file indicating the coordinates of bases covered less than 10X was calculated for each subject, v) In each sample, all wild type homozygotes variants lying in regions covered under 10X were converted to missing data and excluded, vi) for each genetic variant a specific ID was produced combining information of chromosome number, base position, reference base and alternative base (e.g. chr1-542354454-A-T). The pipeline of the framework is available in Supporting Information section.

### Quality controls

A quality control analysis was performed both on samples and genotypes. We calculated the Phred score, a measure of the accuracy of the base calling [31], as Q = -10 log_10_ P, where P is the base-calling error probability. Q<30 means that the probability of having a base-calling error is > 0.001. Genetic variants showing a 1st quartile Phred score < 30 were removed from the pedigree files as well as variants genotyped in less than 98% of samples, variants that resulted not in Hardy Weinberg equilibrium and variants covered under 10X. Samples with a >2% of missing data were also removed. After the quality control step the mean coverage was 95% and the mean depth of coverage was 1130X

### Annotation and classification of genetic variants

The genetic variants were annotated using the software *annovar* [32] and then were classified as common variant if they showed a MAF > 0.05 either in the present dataset or in 1000 genomes, dbSNP or EXAC databases. Conversely they were classified as rare variant if they resulted unknown or showed a MAF < 0.01 in the previously mentioned databases. Genetic variants were also classified as benign, pathogenic and VUS by a team of medical genetics experts and following reported guidelines [33]. For the sake of simplicity and in order to improve statistical power, pathogenic and likely-pathogenic variants were joined and classified as pathogenic.

### Statistical analysis

#### Dimensionality reduction

Dimensionality reduction of the phenotypic traits was obtained by Factor Analysis of Mixed data [34] (FAMD) with the purpose of summarizing the phenotypic variability. The first dimension of the FAMD analysis was used as predictor in a logistic regression model where the presence of at least one pathogenic rare variant on FBN1, TGFBR1 or TGFBR2 genes was considered as dependent variable.

#### Common variant analysis

Associations between common variants and dichotomous phenotypic traits were tested using a logistic regression with age and the number of benign, pathogenic and VUS rare variants as covariates. The max T permutation procedure was performed to correct for multiple testing, using 1000 permutations. For each phenotypic sign, nominally associated variants (unadjusted p value < 0.05) were also selected in order to obtain a Polygenic Risk Score (PRS). The PRS for an individual *i* was defined as

$${PRS}_{I}=w_{1}g_{1i}+w_{2}g_{2i}+\cdots +w_{j}g_{ji}+\cdots +w_{m}g_{mi}$$ (1)

where g_*ji*_ is the number of effect alleles for SNP *j* in individual *i* (taking values of 0, 1 and 2), *w*_*j*_ is the weight for SNP *j* and *m* is the number of variants resulted nominally associated with the phenotypic sign. *w*_*j*_ is defined as *β*_*j*_*/SE(β*_*j*_*)*, where *β*_*j*_ is the per-allele log odds ratio obtained by the logistic model regression and *SE(β*_*j*_*)* is the standard error of *β*_*j*_. This definition of *w*_*j*_ allows to give a greater weight to the SNPs having a more precise estimated effect [35]. The analysis was performed using plink toolset (version 1.07).

#### Rare variants analysis

The effect of rare variants on phenotypic traits was analyzed following two approaches. The first approach investigated their overall impact on the clinical phenotypes of patients. To this aim a negative binomial multiple regression analysis was performed considering the total number of rare variants (both rare and low frequency variants) and the number of clinical manifestations while age was considered as covariate. Following the second approach, the association between phenotypic traits and rare genetic variants was explored at gene-level using the Optimal Sequence Kernel Association (SKAT-O) Test [36]. Boostrap resampling with 1000 resamples was applied in order to obtain more accurate estimates of standard errors. Variants with MAF > 5% were removed before (SKAT-O) Test. Analyses were carried out considering separately the major criteria and the clinical manifestations as outcomes. Clinical classification of rare variants and age were used as covariates. Multiple testing correction was not applied based on three main considerations: multiple comparisons should be considered when interpreting the results rather than in the calculations [37–38], corrections for multiple comparisons are not necessary in exploratory studies that have many preplanned outcomes [39], and corrections for multiple comparisons are not needed when the comparisons are complementary [40] as in this case.

#### Joint analysis of common and rare variants

The combined effect of common and rare variants on each clinical sign were studied by a structural equation model (SEM). PRSs were used for summarizing the common genetic variants’ contribution and for improving the predictive power due to polymorphisms. The number of pathogenic and/or VUS rare variants in the genes analyzed was used to summarize the rare genetic variants’ contribution. Two several SEMs were fitted in order to describe the residual variance of the models. In the first model only age and number of pathogenic/VUS rare variants in the *FBN1* were used as predictors for all the clinical manifestations analyzed. In the second model age, significant results obtained from the rare variants analysis and PRSs obtained from the common variant analysis were used as predictors for all the clinical manifestations analyzed. The first model emulates the classic phenotype-genotype association study that consider only causative mutations in *FBN1* while the second model considers the full genetic variability observed.

A schematic representation of the study design is shown in S1 Fig.

### Software

Associations between the Common variant and the dichotomous phenotypic signs were carried out by the plink toolset (version 1.07). All the other analyses were performed using the R software (version 3.4.4). The *FAMD* function provided in the R package *FactoMineR* was used in order to summarize the complexity of all the phenotypic traits.

Regressions with dichotomous outcomes were fitted using the *glm* function provided in the R package *base*. Regressions with count outcomes were fitted using the *glm*.*nb* function provided in the R package *MASS*. Associations between the rare variants and the phenotypic signs were performed using the *SKAT* function of the *SKAT* R package. Structural equations models were fitted using the *sem* function of the R package *lavaan*.

## Results

### Clinical and phenotypic features of patients

**Fig 1:**
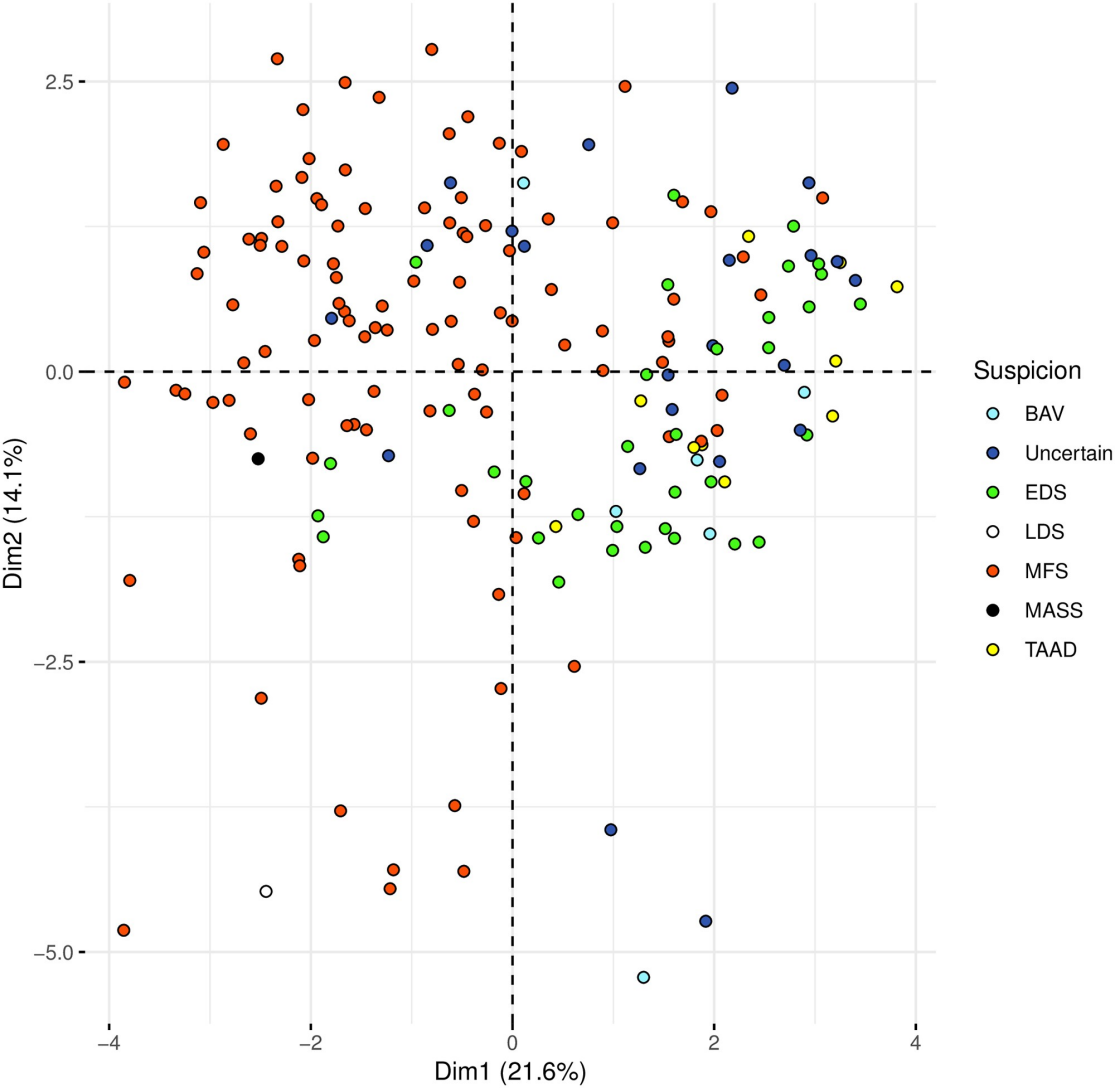
Dimensionality reduction of the phenotypic data and clinical suspicion. The complexity of variability of all the phenotypic traits was reduced using the statistical approach Factor Analysis for Mixed Data (FAMD). Starting from a great number of phenotypic variables, a reduced set of new variables called dimensions were calculated. These new variables are still able to describe the complexity of original phenotypic traits. In particular, the new variables obtained are ordered considering the percentage of variance that they are able to collect. The first two dimensions explain respectively the 21.63% and the 14.06% of the total phenotypic variability. Plots were obtained using the first two dimensions. The first dimension (Dim 1) mainly collects information regarding clinical suspicion of MFS, indeed the majority of the patients showing a clinical suspicion of MFS are closer and have negative values of Dim 1 and are localized on the left part of the panel while samples with a clinical diagnosis of EDS and TAAD, with positive values of Dim 1, are mainly located in the right part of the panel.

Clinical and phenotypic features of the 181 enrolled patients (97 were male and 84 female) were reported in Table 2. The median age± interquartile range (IR) was 39.64 ± 28.66 years. The distribution of phenotypic traits and similarity among subjects were represented in the heatmap plot (S2 Fig). The complexity of variability of all the phenotypic traits was reduced using Factorial Analysis for Mixed Data (FAMD) (Fig 1). Starting from a great number of phenotypic variables we obtained a reduced set of new variables called dimensions, that summarize the information and were still able to describe the complexity of original phenotypic traits. The new set of dimensions obtained were ordered considering the percentage of variance that they were able to collect. Using only the first two dimensions we could describe more than 35% of variance of phenotypes.

**Table 2:** Clinical features of 181 subjects.

**Clinical Suspicion**	**N° of Subjects**	**%**
Marfan Syndrome (MFS)	107	59.13
Ehler-Danlos Syndrome (EDS)	34	18.79
Uncertain	22	12.15
Thoracic Aortic Aneurysm and Dissection (TAAD)	10	5.52
Bicuspid Aortic Valve (BAV)	6	3.31
Loeys-Dietz Syndrome (LDS)	1	0.55
Mass Phenotype (MASS)	1	0.55
**Total**	**181**	**100**
**Clinical signs**	**N° of Subjects**	**%**
Wrist sign	113	62.4
Hyperlaxity	100	55.2
Stretch marks	92	50.8
Arachnodactily	90	49.7
Mitral valve prolapse	86	47.5
Scoliosis	85	47
Flatfoot	83	45.9
Thumb sign	79	43.6
Aortic ectasia	70	38.7
Myopia	64	35.4
Pectus carinatum	33	18.2
Ectopia lentis	28	15.5
Aortic dissection	18	9.9
Reduced elbow extension	13	7.2
**Major Criteria**	**N° of Subjects**	**%**
Cardiac	87	48.1
Ocular	80	44.2
Skeletal	57	31.5
Systemic Score	28	15.5

**Fig 2:**
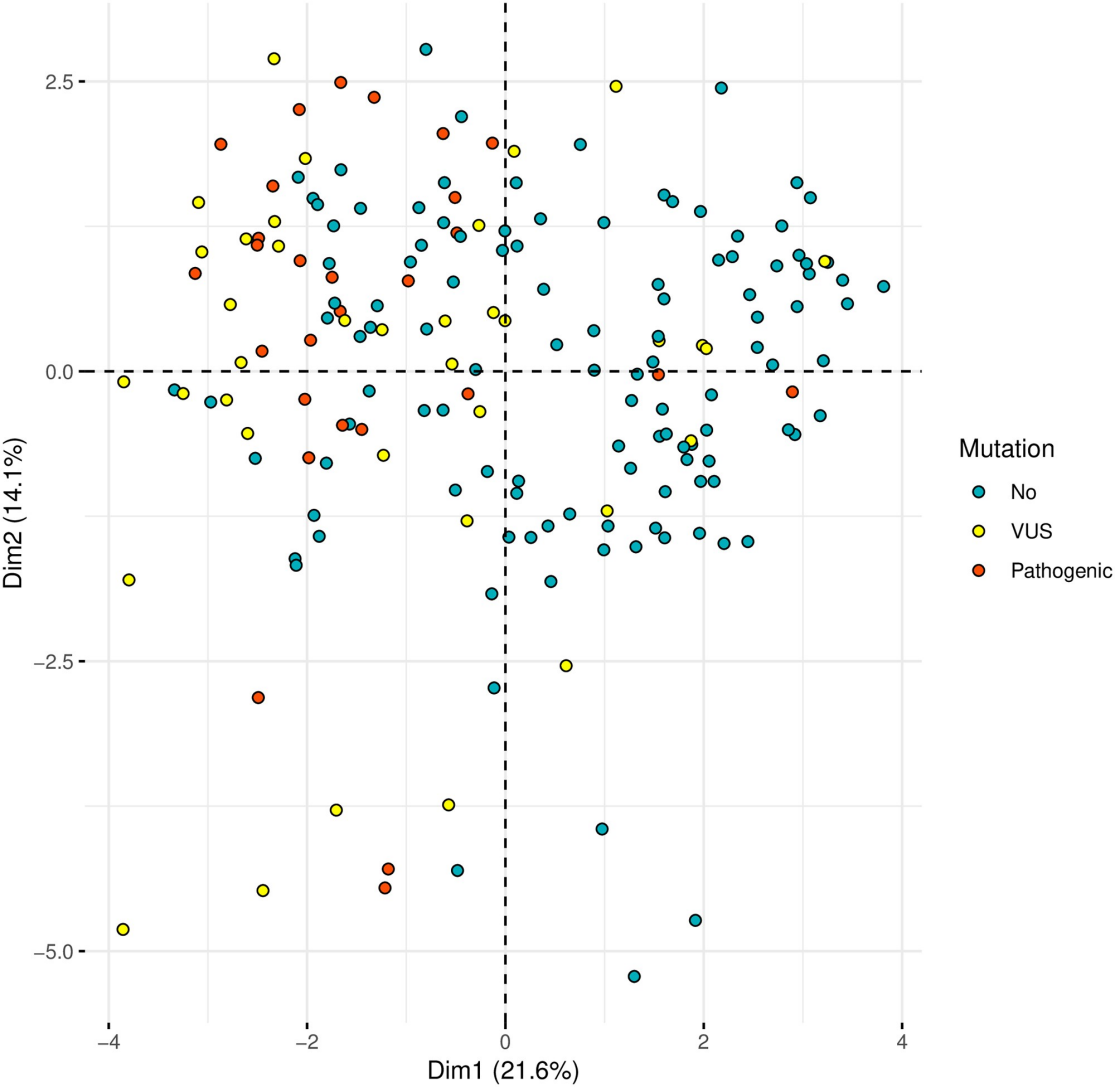
Dimensionality reduction of the phenotypic data and mutation. Dimension reduction obtained by Factor Analysis for Mixed Data (FAMD) is used to visually identified patients with a pathogenic in *FBN1*, *TGFBR1*, *TGFBR2*. Plots were obtained using the first two dimensions. There is a significant association between Dim 1 and the probability to find a mutation in MFS main genes. Indeed subjects carrying a pathogenic variant in *FBN1* or *TGFBR1/2* genes are positioned in the left part of the panel.

The first dimension (Dim 1) mainly collected information regarding clinical suspicion of MFS, indeed the majority of the patients showing a clinical suspicion of MFS are closer and had negative values of Dim 1 and were localized on the left part of the panel while samples with a clinical diagnosis of EDS and TAAD, with positive values of Dim 1, were mainly located in the right part of the panel. A logistic regression model was fitted in order to evaluate if Dim 1 was associated with the presence of any pathogenic rare variant in MFS main genes (i.e., *FBN1*, *TGFBR1* or *TGFBR2*). Dim 1 was significantly associated with the presence of at least one pathogenic variant in one of the genes analyzed (OR = 1.89, 95% C.I. 1.542–2.376). Fig 2 shows that patients carrying a pathogenic variant in *FBN1* or *TGFBR1/2* genes are positioned in the left part of the panel.

### Genetic variability

**Fig 3:**
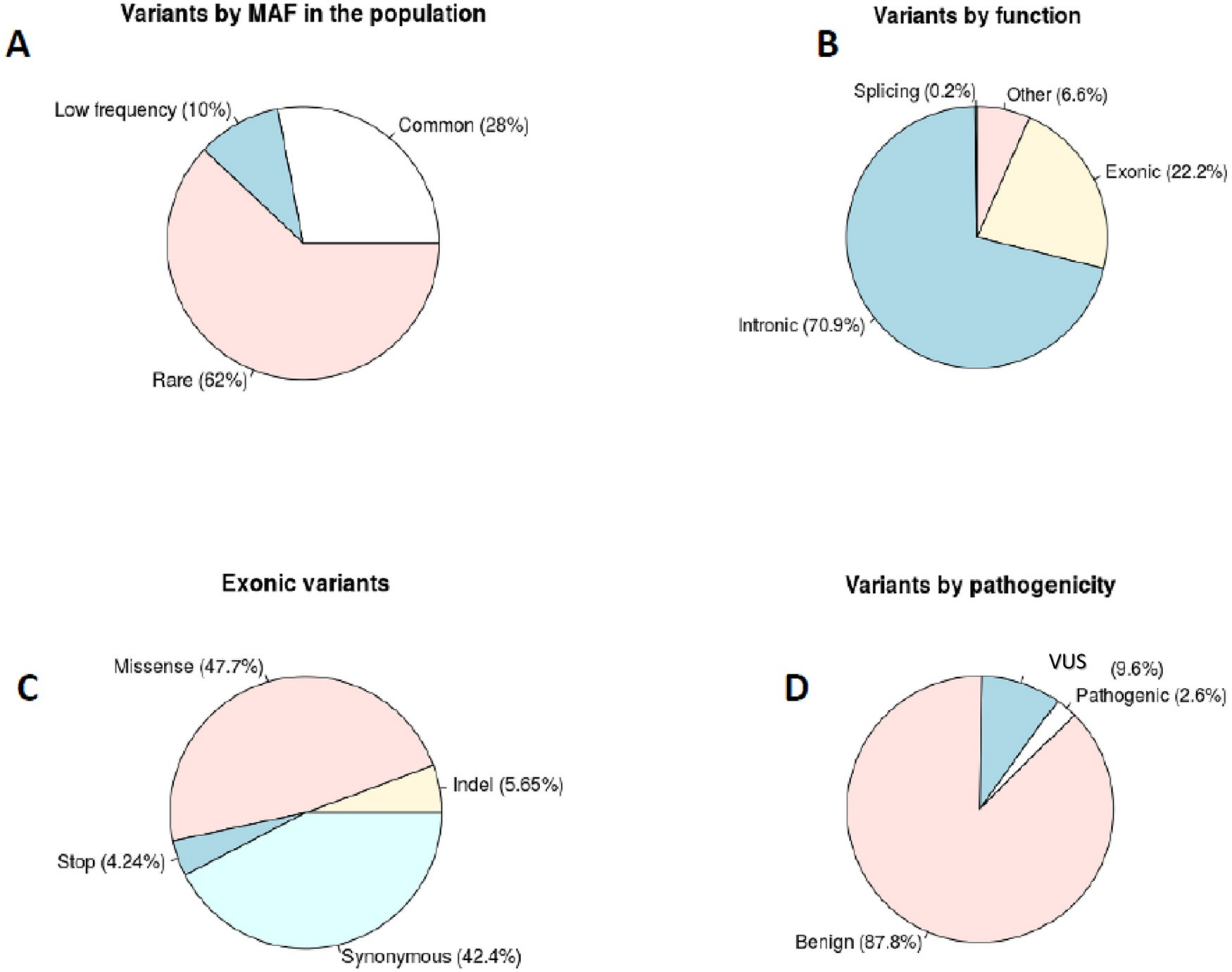
Description of the genetic variability. Distribution of genetic variants considering Minor Allele Frequency (MAF) (A), considering their genomic function (B), based on exonic type (C), considering their clinical classification (D).

The NGS analysis of the 11 candidate genes in 181 unrelated samples identified 361 (28%) common variants (MAF > 0.05), 128 low frequency variants (10%) (0.01 < MAF < 0.05) and 784 rare variants (62%) (MAF < 0.01) Fig 3A. Among these 1273 genetic variants 283 were exonic (22.2%), 903 were intronic (70.9%), 3 were splicing variants (0.2%) and 84 were localized in UTR regions (6.6%) Fig 3B. Exonic variants were mostly missense (47.7%) and synonymous (42.4%) while stop variants and indels were 4.2% and 5.6% respectively Fig 3C. Based on standards and guidelines all the variants were also classified as benign (87.8%) pathogenic (2.6%) (including also likely-pathogenic) and VUS (9.6%). Results are shown in Fig 3D. Additional annotation data were generated using annovar software for the subset of pathogenic (including also likely-pathogenic) variants. They included: the location of the mutation in the protein domain, MAF from several public database (1000G, EXAC, ESP, gnomAD), clinical classification from Clinvar database, pathogenicity predictions obtained with several tools (SIFT,Polyphen2, LRT, MutationTaster, MutationAssessor, FATHMM, PROVEAN) all results are shown in S1 Table.

### Common variant association analysis

Common variant association analysis was investigated via a logistic regression with each clinical sign in turn as outcome and each genetic variant with a MAF > 0.05 as covariate, adjusting by age and the number of benign, pathogenic and VUS rare variants. After multiple testing correction a common variant of *COL1A1* gene, rs2075557, was associated with scoliosis (OR = 0.2533, adjusted p-value = 0.04), moreover other 4 SNPs in *COL1A1* were also associated with scoliosis, although at marginal level: rs2696247 (OR = 0.266, adjusted p-value = 0.07), rs2075554 (OR = 0.267, adjusted p-value = 0.102), rs2277632 (OR = 0.295, adjusted p-value = 0.121), rs2586494 (OR = 0.305, adjusted p-value = 0.141). After multiple testing correction, several common variants failed to reach significant association levels, however they were nominally associated (unadjusted p value < 0.05) to several phenotypic signs and they were reported in S2 Table. A representative Fig. with haplotypes of common variants resulted associated with phenotypic traits was shown in S3 Fig. Common variants resulted nominally associated were also evaluated and tested toward a reference collective. To this aim we obtained variants frequencies dataset from gnomAD database. All the associations observed were replicated using this second reference collective. Results of this second analysis were also reported in S2 Table. Furthermore, the effect of common variants on phenotypic traits was tested in the subset of subjects who clearly met Ghent criteria. No common variants resulted statistically significant after multiple testing correction although a set of common variants resulted still nominally associated with traits investigated. Results are shown in S3 Table

### Rare variants association analysis

The effect of rare variants on phenotypic traits was tested following two strategies. The first strategy investigated their overall impact on the clinical phenotypes. This analysis showed that the overall number of rare genetic variants is significantly associated with the number of clinical manifestations (RR = 1.018, p-value = 0.018). Based on this result, the same regression model was used to investigate the role of rare variants considering also their clinical classification (i.e, pathogenic, VUS and benign). Only the number of pathogenic and VUS rare variants resulted significantly associated with the number of clinical manifestations (RR = 1.533, p-value = 9.87e-06; RR = 1.045, p-value = 0.024 respectively). As to the second strategy, the association between phenotypic traits and rare genetic variants was explored at gene-level via SKAT-O and using age as covariate. This analysis confirmed that pathogenic and VUS rare variants have a significant impact on several clinical manifestations while benign rare variants were not significantly associated with any of the phenotypic traits considered. Moreover, for several phenotypic traits a polygenic contribution of rare variants was also observed. In particular, aortic ectasia resulted significantly influenced by *FBN1* rare pathogenic variants and by *FBN1*, *COL1A1* and *COL3A1* VUS, arachnodactyly resulted influenced by *FBN1* rare pathogenic and VUS rare variant; *MYH11* pathogenic variants and FBN1 VUS resulted associated with ectopia lentis. The mitral valve prolapse appeared to be significantly influenced by both pathogenic and VUS rare variants in *FBN1* and by *NOTCH1* and *TGFBR2* VUS. VUS of *ACTA2* emerged as significantly associated with aortic dissection while *COL1A1* and *COL1A2* VUS significantly influenced hyperlaxity. Other phenotypic traits such as scoliosis, flat foot, wrist sign, pectus carinatum were associated only with pathogenic or VUS variants in *FBN1*. Significant results obtained for each clinical sign and major criteria, both for pathogenic and VUS variants are reported in S4 Table. Also for rare variants the analysis was performed considering as reference the collective obtained from gnomAD database. Results confirmed a great part of associations previously observed using internal controls and were reported in S4 Table. The effect of rare variants on phenotypic traits was also tested in the subset of subjects who clearly met Ghent criteria. This analysis confirmed the polygenic effect of pathogenic and VUS rare variants on phenotypic signs, although in this analysis other genes emerged as statistically significant. Results are shown in S5 Table.

### Rare and common variant combined effect analysis

**Fig 4:**
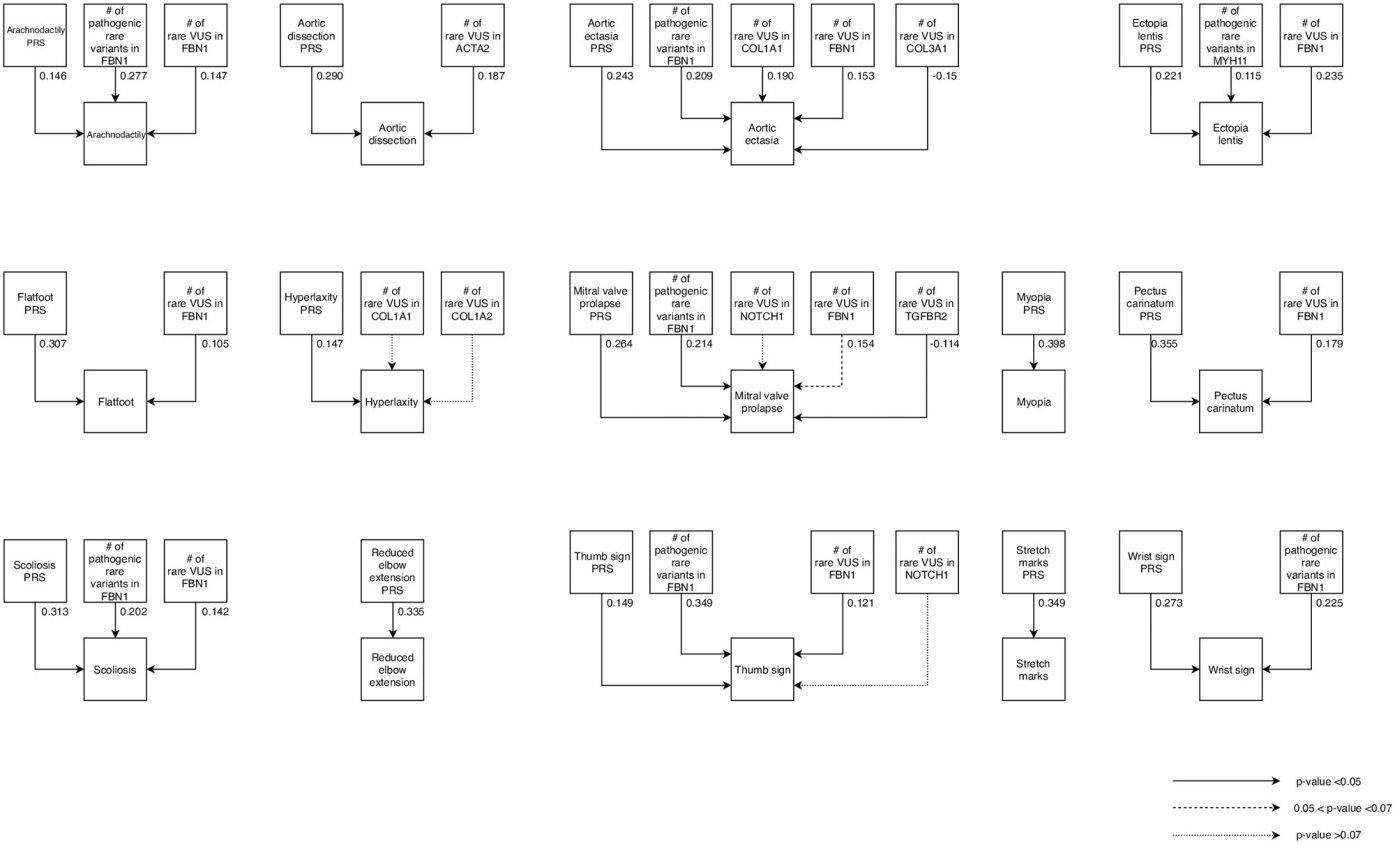
Graphical representation of structural equation model analysis. For each phenotypic trait the effects of common variant (PRS) and rare variants, that resulted associated from previous analyses, were combined together.

The combined effect of common and rare variants on each clinical sign was explored by SEM. For each phenotypic trait, results obtained from the common variant analysis were firstly collected in sign specific PRSs as described in materials and method section. PRSs and significant results obtained from the rare variant analysis were finally used as exogenous variables in the SEM. Fig 4 shows the model we proposed. This analysis showed that several phenotypic traits are significantly influenced by both common and rare variants. Scoliosis, arachnodactyly, aortic ectasia, mitral valve prolapse, thumb sign, ectopia lentis, wrist sign, aortic dissection, flatfoot, pectus carinatum, were significantly associated both with common variants and with the number of pathogenic or VUS variants. Conversely a number of clinical manifestations such as hyperlaxity, myopia, reduced elbow extension and stretch marks were significantly associated with common variant scores only (Fig 4). Results obtained combining both common and rare variants contributions (model 2) were finally compared to those obtained considering only the influence of a pathogenic and VUS variants in *FBN1* (model 1). Age was considered as covariate both in model 1 and 2. Table 3 reports the percentages of explained variance among the two models. For all the phenotypic traits considered in the study, the amount of explained variance was significantly greater when considering both common and rare variants contributions together (model 2). The mean increase was 11% (p value = 1.6.10–7).

**Table 3:** Percentage of explained variance among the different SEM models. For each phenotypic sign Model 1 considers only age and pathogenic or VUS rare variants on FBN1 gene. Model 2 considers age, pathogenic or VUS rare variants resulted from the SKAT-O test analysis and the sign specific polygenic risk scores.

Clinical signs	Model 1 (%)	Model 2 (%)
Arachnodactily	20.1	25.5
Aortic dissection	0	13.4
Aortic ectasia	8.8	21.2
Ectopia lentis	9.2	15.6
Flatfoot	11.3	21.4
Hyperlaxity	0	18.3
Mitral valve prolapse	5.2	14.8
Myopia	0	15.8
Pectus carinatum	3.6	16.6
Reduced elbow extension	0	15.7
Stretch marks	0	12.2
Scoliosis	5.9	14.5
Thumb sign	17.6	21.9
Wrist sign	8	17.8
**Positivity of Ghent criteria**	**24.8**	**26.6**

## Discussion

In the present study the genotype-phenotype correlation was investigated in a bidirectional way: i) considering the possibility to predict presence of a pathogenic variant in MFS main genes based only on the analysis of clinical signs, and ii) the possibility to improve our knowledge about clinical traits variability and evolution of the disease based on an extended genetic characterization of subjects. Results obtained indicate that information about all phenotypic traits can be summarized in a new variable that resulted significantly associated with the probability to find a pathogenic variant in MFS main genes (FBN1 and TGFBR1/2). This is very important since the choice of the genetic test is often influenced by a correct clinical classification of patients. Moreover, the genotype-phenotype correlation was extended to the overall common and rare genetic variability obtained by NGS analysis. The strategy to combine both rare and common genetic variant effects in an inclusive model allowed to increase the percentage of phenotypic variability that we were able to explain based only on genetic factors and highlighted the opportunity to take advantage of genetic information and NGS data to have a better clinical classification of patients.

The investigation of genotype–phenotype correlation in Marfan-like *habitus* can be considered a challenge given the age-related and pleiotropic nature of the Marfanoid spectrum, its heterogeneity as well as the overlap with other syndromic conditions. Despite significant progress in the understanding of the molecular defects underlying Marfan spectrum, most published researches have only identified moderate genotype-phenotype correlations [41]. Some studies suggested that pathogenic variants laying between exons 24 and 32 are associated with more severe marfanoid presentations [42], other studies found an enrichment of a specific subtype of missense variants (cysteine substitutions) in MFS patients with ectopia lentis [43]. The FBN1 protein contains a number of repeated structural modules, mostly represented by the epidermal growth factor-like calcium-binding domain (EGF_CA). Biochemical evidences show that these cysteine residues are necessary for proper folding and calcium binding of the EGF_CA module, which in turn participates in proper secretion and deposition of FBN1 [44]. In a couple of studies, aortic dissection appeared to be more common in subjects carrying a premature termination variant, compared with individuals having a cysteine substitution [41,45]. In addition, a large genotype–phenotype study reported also that patients with a stop codon variant more frequently had skeletal and skin involvement. Notwithstanding these results, the genotype-phenotype correlation in MFS and Marfan-like disorders still remain largely to be explored. It should be noted that all the studies proposed to date mainly focused their attention on *FBN1* pathogenic variants and, based on molecular, functional, structural, clinical classification of these variants, explored correlations with phenotypic traits. The present study aimed to improve the genotype-phenotype correlation by widening the amount of genetic information used. To this aim the molecular analysis was extended by NGS considering also other genes (i.e., *TGFBR1*, *TGFBR2*, *COL1A1*, *COL1A2*, *COL3A1*, *COL5A1*, *COL5A2*, *MYH11*, *NOTCH1* and *ACTA2)* that are known to play an important role in connective tissue development, Marfan-like syndrome and marfanoid spectrum. The genotype-phenotype association analysis was performed by examining the extended genetic information and not only the putative pathogenic or likely-pathogenic variants, in a bidirectional way with the aims to:

1. predict the presence of a potential pathogenic variants in MFS known genes (e.g. *FBN1* or *TGFBR1/2*) based only on the analysis of clinical manifestations.
2. obtain a better comprehension of phenotype variability through a deeper genetic characterization and investigating both common and rare genetic contribution.

For the first goal the complexity of variability of all the phenotypic traits was reduced using a statistical approach called FAMD. Starting from a great number of phenotypic variables we obtained a reduced set of new variables called dimensions, that were still able to describe the complex variance of original phenotypic traits. The new variables obtained resulted ordered considering the percentage of variance that they were able to collect. Using only the first two dimensions we could describe more than 35% of variance of all phenotypic traits. In particular way the first dimension resulted associated with clinical classification of patients: subjects suspected to be Marfan were localized on the left part of the panel while samples with a clinical picture of EDS and TAAD were mainly restricted to the right part. Based on this finding we investigated the potential association between the first dimension and the presence of a pathogenic or likely-pathogenic variants in Marfan main genes (*FBN1* and *TGFBR1/2*). The result confirmed the hypothesized association supporting the possibility to predict the presence of a pathogenic or likely-pathogenic variant associated with MFS, based only on a phenotypic characterization of patients. In particular, the reported association indicates that the probability to find a pathogenic or likely-pathogenic variant in *FBN1 or TGFBR1/2* increases of 90% for each unit decrease of Dim 1. This result is suggestive but it could not be considered completely a new finding since it is known that a pathogenic variant in *FBN1* is expected in 82–83% of subjects who met 2010 revised Ghent criteria [46,47]. However, the main novelty of this approach is represented by the possibility of obtaining a continuous variable that might be useful to estimate the exact probability of finding a pathogenic variant also in subjects who don’t meet 2010 revised Ghent criteria. In fact, we were able to predict the presence of a pathogenic variant in 4 subjects (characterized by Dim 1 values smaller than—2.0) which really had a confirmed pathogenic variant in *FBN1* notwithstanding they were negative for 2010 revised Ghent criteria. Results in Fig 2, show also that pathogenic variants were mainly localized on the left of the panel, suggesting a possible stratification among pathogenic and VUS rare variants. This observation leads to speculate that the strategy, adopted to analyze phenotypic traits in Marfan spectrum, might be also useful to improve clinical classification of rare genetic variants. In other words if there is a significant association between Dim 1 and the presence of a pathogenic variant in Marfan main genes, basing on phenotypic stratification of a given subject we can for example calculate an *a priori* probability that a given FBN1 VUS found in that patient might be pathogenic. To date, the pathogenicity status of the rarest variants still remains unknown and this represents an important challenge in clinical genetics. Furthermore, we can also speculate that subjects with extremely negative values of Dim 1 (in which we expect the presence of a pathogenic or likely-pathogenic variant) but resulted negative at genetic test, should be considered candidate patients for a copy number analysis in *FBN1* or *TGFBR1/2*.

This study represents a proof of concept that demonstrated how a model able to consider a wide array of genetic information can improve the genotype-phenotype correlation. However some limitations have to be declared: the reduced amount of phenotypic data available and the number of subjects analyzed weakened considerably the power of this study. However, this study aimed to explore new strategies useful to investigate genotype-phenotype correlation and under this perspective, results reported herein appear to be promising.

In order to obtain a better comprehension of phenotype variability we extended the genetic analysis to other genes already known to be associated with Marfan-like disorders and considered also the entire genetic variability observed and not only pathogenic or likely pathogenic variants of FBN1. To this aim we developed a bioinformatic framework able to organize NGS results in a uniformed genotypic dataset (The software code of this framework is available in **Supplementary Materials**).

For all the variants with a MAF > 0.05 a classical association analysis was performed. This analysis identified a group of SNPs of the *COL1A1* gene having a protective effect towards scoliosis. This is an interesting finding because these genetic variants have never been reported before to be associated with scoliosis although several pathogenic variants of *COL1A1* were previously associated with this spine abnormality [48,49].

For each phenotypic trait, the association analyses identified several common variants that were nominally associated. Considering the reduced power of this study these variants were used to obtain sign-specific PRSs. Polygenic scores have recently been used to summarize genetic effects among an ensemble of markers that do not individually achieve significance in a large-scale association study [50]. We adopted this strategy in order to summarize for each phenotypic sign the effect of the genomic background (defined as the sum of common genetic variants) [51]. The exploratory analysis of genetic variability revealed that the greater part of the genetic variants corresponds to rare (62% with a MAF<0.01) or low frequency variants (10% with a 0.01 < MAF < 0.05). This observation leads to speculate that the contribution of these variants to phenotype variability should not be considered marginal and that genotype-phenotype correlation analyses should take into consideration their role.

The effect of rare variants on clinical phenotypes was explored firstly considering their overall impact, highlighting a significant association between the number of clinical manifestations and the number of rare variants. This interesting finding confirms that each rare variant has an effect that contributes to increase the complexity of the phenotypic picture.

The same association was also tested classifying all the rare variants following indication proposed by ACMG guidelines as benign, pathogenic, likely-pathogenic and VUS. For statistical power reason pathogenic and likely-pathogenic variants were joined together and considered as pathogenic. The number of pathogenic and VUS variants resulted significantly associated with the number of clinical manifestations and, as expected, the effect for pathogenic variants was greater than that observed for VUS.

Gene-level association between each phenotypic traits and rare genetic variants was analyzed using the SKAT-O test, considering both the gene involved and the clinical classification of the rare variants. This analysis highlighted that also rare variants contribute in a polygenic manner to the phenotype characterization.

The same analysis was replicated in a subgroup of subjects who clearly met the Ghent criteria. This secondary analysis confirmed the polygenic nature of the effect of rare variants on the phenotype confirming some previous results. Furthermore it should be noted that in this cohort all the subjects were phenotypically similar and had at least one VUS or a pathogenic variant in FBN1, this led to unmask some effects of other genes that in the previous analysis were not statistically significant after multiple correction. It can be speculated that these new associations failed to emerge in the main analysis probably because they were masked by the strongest effect of the rare FBN1 variants.

Effects that rare and common variants exert on phenotypic traits were firstly estimated separately, but then results obtained were joined in order to achieve, for each phenotypic trait, an estimation of the variance explained. Lastly, we compared the amount of variance explained by this approach to the percentage of variance explained by a model that considers only VUS or pathogenic variants in *FBN1* gene (this last model aimed to mimic the approach used by previous studies). In this paper we compared a model of genotype-phenotype association analysis that considers only the clinically relevant variant (like previous studies) to a new model that considers all the genetic variants identified by NGS. We compared the two models evaluating the percentage of variance that each model could explain. For all the phenotypic traits studied the model of genotype-phenotype correlation analysis proposed herein (Model 2) resulted able to explain a greater amount of phenotypic variance showing a significant mean increase of 11%. These results are suggestive and support the idea that we need to consider the full complexity of genetic information when doing a genotype-phenotype correlation. Moreover, it has to be aware that in last decade NGS methods allowed to obtain a greater amount of genetic information that is not still generally used in its entirety. Although, these results are promising, there are several limitations previously mentioned that have to be considered. i) The genetic analysis was extended to 11 genes that are known to play a role in connective tissue diseases although they can be considered only a reduced list of representatives. ii) The same limitation can also be considered for phenotypic data available for this study that are limited. A gene panel including a broader list of candidate genes as well as a better and thorough phenotypization of patients might improve considerably results that could be obtained. This study might have relevant clinical implications. Starting from several phenotypic traits we were able to synthetize them in a new variable useful for clinical classification of patients. Moreover, basing on this variable we obtained an a priori probability of finding a pathogenic mutation in Marfan main genes. In addition, considering one subject carrying a VUS in one of Marfan main genes, looking at his dim 1 values we can estimate an a priori probability useful to improve clinical classification of that genetic variant. Finally, mutation negative subjects, showing values of dim 1 that support an involvement of FBN1 or TGFBR1/2 with a very high risk of having a pathogenic variant in these genes, might be considered candidates for CNV analysis of these genes. In conclusion, the strategy to combine both rare and common genetic variants in an inclusive model appeared to be a winning solution in order to improve the knowledge regarding phenotypic and clinical variability. This is considered a central problem in the field of personalized medicine and results obtained herein lead to speculate that we are not so far from this goal.

## Supporting information

S1 Fig Representative description of study design. (TIF)

S2 Fig Heatmap plot representing the distribution of phenotypic traits and similarity among subjects. For each subject presence of a phenotypic traits is indicated in yellow while absence of the trait is indicated in blue.
(TIF)

S3 Fig Phenotypic traits and haplotypes. A representative Figure indicating for each phenotypic trait the haplotypes of common dbSNP variants resulted nominally associated.
(TIF)

S1 Table Additional annotation data. For the subset of pathogenic (including also likely-pathogenic) variants filter based annotation was made using the software annovar. An extensive description of each column and database used can be found at the link: http://annovar.openbioinformatics.org/en/latest/user-guide/filter/.
(XLSX)

S2 Table Common variants resulted nominally associated (unadjusted p value < 0.05) to several phenotypic signs. Results were obtained using both internal controls and data from gnomAD database as reference population. For SNVs that are not present in gnomAD database results are indicated as NAs.
(XLSX)

S3 Table Common variant nominally associated in Ghent positive cohort. Common variant that failed to reach significant association levels, however they resulted nominally associated (unadjusted p value < 0.05) to several phenotypic signs in the Ghent 2010 positive cohort.
(XLSX)

S4 Table Optimal Sequence Kernel Association Test (SKAT-O) significant results. Significant results of the SKAT-O test by pathogenic (A) and VUS (B) rare variants for major criteria and clinical signs.
(XLSX)

S5 Table Optimal Sequence Kernel Association Test (SKAT-O) significant results for Ghent positive subjects. Significant results of the SKAT-O test by VUS rare variants for major criteria and clinical signs for the subjects who are positive to Ghent Criteria.
(XLSX)

S1 Text PIPELINE_PEDIGREE.txt. The code that produces a Plink formatted pedigree files starting from a folder containing all VCF files.
(TXT)
