# Supplementary figures and images for: NGS analysis in Marfan syndrome spectrum: Combination of rare and common genetic variants to improve genotype-phenotype correlation analysis
Source: PLoS One. 2019 Sep 19;14(9):e0222506. doi: 10.1371/journal.pone.0222506 (PMC6752800; doi:10.1371/journal.pone.0222506)

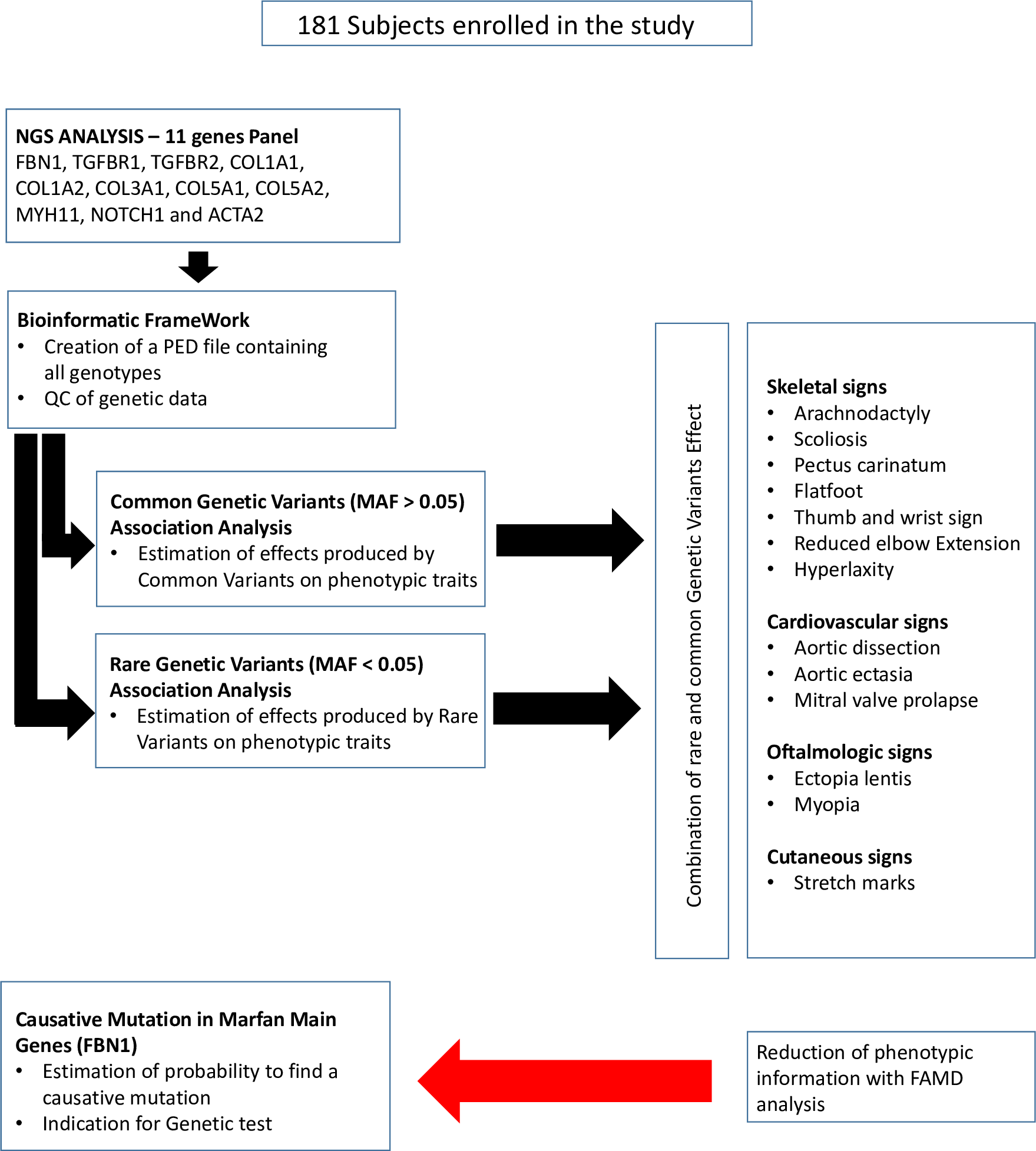

Supplement: S1 Fig — (TIF) [file pone.0222506.s001.tif]

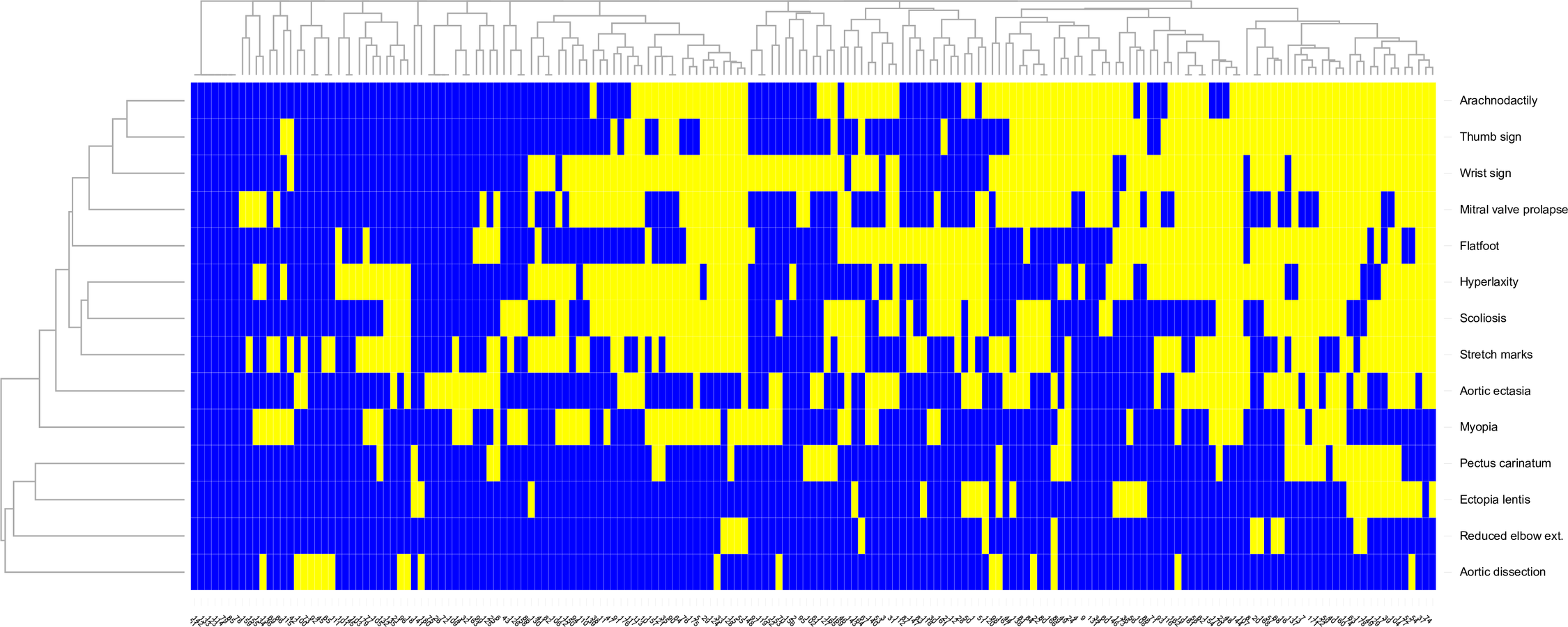

Supplement: S2 Fig — For each subject presence of a phenotypic traits is indicated in yellow while absence of the trait is indicated in blue. (TIF) [file pone.0222506.s002.tif]

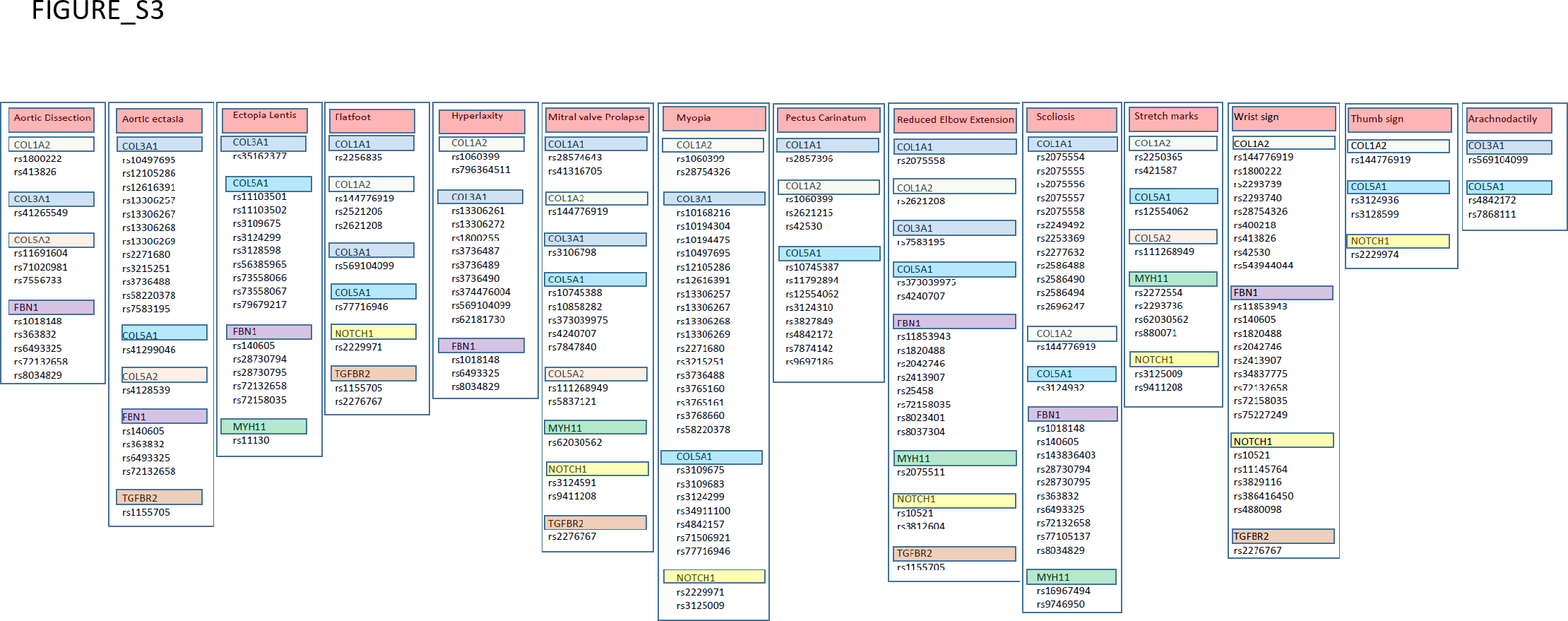

Supplement: S3 Fig — A representative Figure indicating for each phenotypic trait the haplotypes of common dbSNP variants resulted nominally associated. (TIF) [file pone.0222506.s003.tif]
